# A nucleic acid detection assay combining reverse transcription recombinase-aided amplification with a lateral flow dipstick for the rapid visual detection of porcine deltacoronavirus

**DOI:** 10.1080/21505594.2022.2116157

**Published:** 2022-09-05

**Authors:** Jianyu Zeng, Wenlong Wang, Lei Zhou, Xinna Ge, Jun Han, Xin Guo, Yanhong Chen, Yongning Zhang, Hanchun Yang

**Affiliations:** Key Laboratory of Animal Epidemiology of Ministry of Agriculture and Rural Affairs, College of Veterinary Medicine, China Agricultural University, Beijing, P.R. China

**Keywords:** Detection, lateral flow dipstick, nucleic acid, porcine deltacoronavirus, recombinase-aided amplification

## Abstract

Porcine deltacoronavirus (PDCoV) is an emerging enteropathogen causing severe diarrhoea, dehydration, and death in nursing piglets and enormous economic losses for the global swine industry. Furthermore, it can infect multiple animal species including humans. Therefore, a rapid, definitive diagnostic assay is required for the effective control of this zoonotic pathogen. To identify PDCoV, we developed a nucleic acid detection assay combining reverse transcription recombinase-aided amplification (RT-RAA) with a lateral flow dipstick (LFD) targeting the highly conserved genomic region in the *ORF1b* gene. The RT-RAA-LFD assay exhibited good PDCoV detection reproducibility and repeatability and could be completed within 11 min. Ten minutes at 40 °C was required for nucleic acid amplification and 1 min at room temperature was needed for the visual LFD readout. The assay specifically detected PDCoV and did not cross-react with any other major swine pathogens. The 95% limit of detection (LOD) was 3.97 median tissue culture infectious dose PDCoV RNA per reaction. This performance was comparable to that of a reference TaqMan-based real-time RT-PCR (trRT-PCR) assay for PDCoV. Of 149 swine small intestine, rectal swab, and serum samples, 71 and 75 tested positive for PDCoV according to RT-RAA-LFD and trRT-PCR, respectively. The diagnostic coincidence rate for both assays was 97.32% (145/149) and the kappa value was 0.946 (p < 0.001). Overall, the RT-RAA-LFD assay is a user-friendly diagnostic tool that can rapidly and visually detect PDCoV.

## Introduction

Porcine deltacoronavirus (PDCoV) is a novel enteropathogenic coronavirus causing severe vomiting, diarrhoea, and dehydration as well as mortality, especially in nursing piglets [1–3]. It belongs to the genus *Deltacoronavirus* within the *Coronaviridae* family [1,4]. The PDCoV genome consists of a single-stranded, positive-sense RNA ~25 kb in length that encodes the 5'untranslated region (5' UTR), the open reading frame 1a/1b (ORF1a/1b), the spike (S), envelope (E), and membrane (M) proteins, the nonstructural protein 6 (NS6), the nucleocapsid (N), the nonstructural protein 7 (NS7), and the 3'untranslated region (3' UTR) [1,2].

PDCoV was initially discovered in swine rectal swabs sampled during a 53-mo molecular epidemiological survey performed in Hong Kong, China [4]. However, the pathogenicity of PDCoV to domestic pigs was not recognized until 2014 when its genome was detected in intestinal and faecal samples of diarrhoeic pigs in Ohio, USA [5]. At nearly the same time, PDCoV was successfully isolated from the intestinal contents of diarrhoeic pigs [3]. Subsequent challenge experiments confirmed that PDCoV is an important causative agent of diarrhoea in pigs and particularly in neonates [6,7]. Outbreaks of pig diarrhoea caused by PDCoV were then reported in Canada, Korea, China, Japan, Vietnam, Thailand, Laos, Mexico, and Haiti [1,8,9] and caused considerable economic losses to the global pig industry [2]. Birds, chickens, turkey poults, and calves could also become PDCoV hosts under natural and experimental conditions [10,11]. PDCoV can infect a wide range of cell lines derived from pigs, humans and chickens [12]. The foregoing evidence suggests that PDCoV poses the potential risk of cross-species transmission [8,12,13]. A 2021 study reported that PDCoV was successfully isolated from the plasma of three Haitian children presenting with an acute undifferentiated febrile illness [9]. Hence, there may also be a public health risk associated with PDCoV.

In pigs, PDCoV infection has a complex clinical manifestation. It may occur as a single infection, co-infection, or multiple infections [8,14]. Co-infection of PDCoV with one or two other swine viral enteropathogens such as porcine epidemic diarrhoea virus (PEDV), porcine enteric alphacoronavirus (PEAV), transmissible gastroenteritis virus (TGEV), and porcine rotavirus (PRoV) is fairly common in pig herds worldwide and significantly aggravates existing diarrhoea [1,8,15]. The clinical signs and pathological features of the foregoing swine diarrhoeal diseases are often indistinguishable [16–18]. Thus, rapid, reliable differential diagnostic methods for PDCoV are required to improve countermeasures against it.

Traditional and novel diagnostic methods have been established and applied to diagnose PDCoV. These include SYBR green-based real-time RT-PCR [19], TaqMan-based real-time RT-PCR (trRT-PCR) [20], microfluidic real-time reverse transcription loop-mediated isothermal amplification (RT-LAMP) chips [21], indirect or double antibody sandwich enzyme-linked immunosorbent assay (ELISA), and fluorescent microsphere immunoassay [22,23]. Nevertheless, the foregoing techniques require highly specialized equipment, trained, experienced personnel, and time-consuming diagnostic processes. Hence, their widespread clinical application is limited. Therefore, rapid, simple, visual diagnostic methods with on-site detection potential are needed.

**Figure 1.**
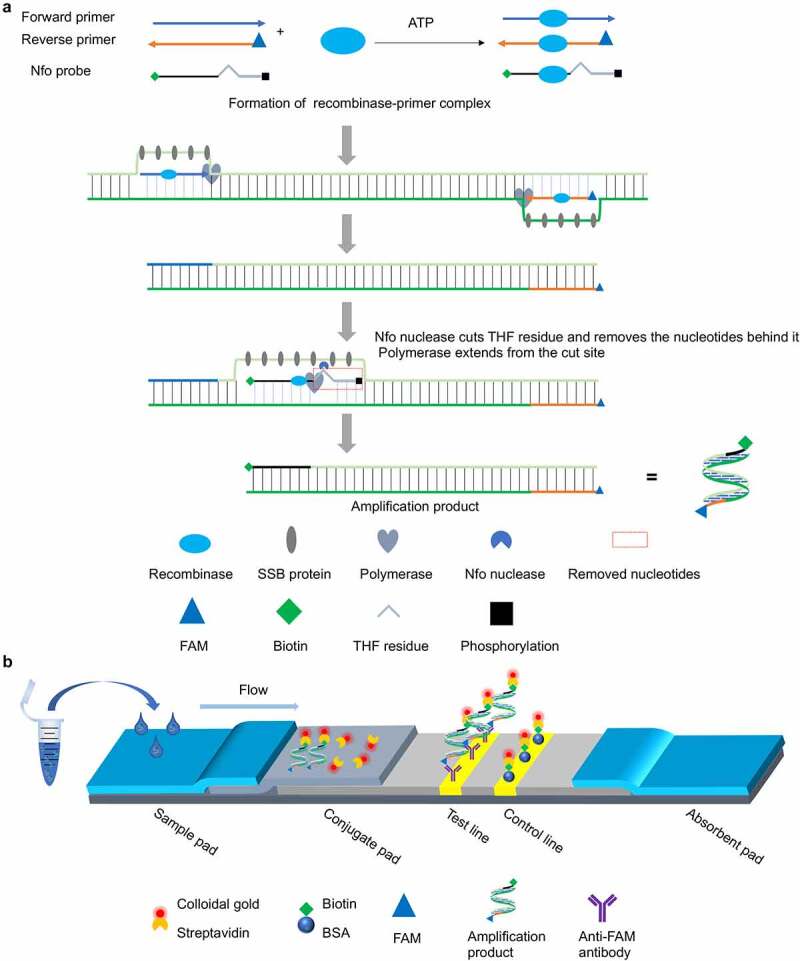
**Schematic of the RT-RAA-LFD assay developed for rapid visual detection of PDCoV nucleic acids**. (a) Schematic diagram of the working principle of the nfo probe-based RT-RAA assay for PDCoV nucleic acid amplification. ATP-dependent recombinase binds each single-stranded oligonucleotide to form recombinase-primer/probe complexes that scan the duplex RNA template and recognize their respective homologous sequences. Upon homology detection, the primers and nfo probe hybridize to the target sequence through strand exchange and initiate DNA synthesis from the 3ʹ end under the action of DNA polymerase. The competitive binding regions in the primers and nfo probe within the displaced template strand are embedded by single-stranded DNA-binding (SSB) proteins to impede re-hybridization to the complementary chain. When the nfo probe hybridizes with the complementary strand, nfo nuclease recognizes and cuts the tetrahydrofuran (THF) residue in the probe and causes the blocking phosphate group behind THF to dissociate. Thence, a fresh 3ʹ-hydroxyl group is generated and it serves as a priming site for DNA polymerase extension. The cleaved probe and the reverse primer eventually form double-stranded amplicons integrating the antigenic labels biotin and FAM (6-carboxyfluorescein) at both 5’ termini, respectively. (b) Schematic of the sandwich-type lateral flow dipstick for detection of the PDCoV-specific amplicons produced by the nfo probe-based RT-RAA assay. The PDCoV-positive amplicons bearing the FAM and biotin antigenic labels flow through the conjugate pad pre-coated with colloidal gold-labelled streptavidin. The FAM-biotin-integrated amplicons bind the colloidal gold-labelled streptavidin and form a complex. The complex flows through the NC membrane and the anti-FAM monoclonal antibody fixed on the test line captures the complex via specific reactions with it. In this manner, a sandwich structure forms and it exhibits a characteristic red band indicating a positive result. The colloidal gold-labelled streptavidin and any excess free complex (if formed) flow forward and are captured by the bovine serum albumin (BSA)-biotin conjugate immobilized on the control line via specific reactions between the biotin and the colloidal gold-labelled streptavidin. In this way, a second red band is generated and the test results are validated. The appearance of red bands at both the test and control lines indicates positive detection. In contrast, the appearance of a single red band at the control line suggests a negative result.

Recombinase-aided amplification (RAA) is an improved version of the recombinase polymerase amplification (RPA) isothermal amplification technique. It has been optimized for the rapid diagnosis of human and animal infectious diseases [24–26]. RAA has been successfully implemented for the rapid detection of African swine fever virus which also seriously threatens the global pig industry [26]. RAA has also been applied to diagnose severe acute respiratory syndrome coronavirus-2 which has endangered human health worldwide [25]. RAA depends mainly on a recombinase, a single-stranded DNA-binding protein, and a strand-displacing DNA polymerase [27]. The general working principles of RAA are illustrated in Figure 1(a) and described in detail in the figure legend. Overall, RAA is more cost-effective than RPA and yet it retains all of its technical advantages [28]. Within <20 min, it can exponentially amplify target genes within 37–42 °C [24]. Rapid diagnosis of an infectious disease also requires rapid, reliable interpretation of the amplification products. Lateral flow dipstick (LFD) technology is a portable auxiliary tool that has been successfully applied in nucleic acid-, antigen-, and antibody-based diagnostics and its results are directly visible to the unaided eye [29]. Thus, LFD has on-site detection potential.

To provide a rapid, visual and user-friendly nucleic acid detection method that can detect PDCoV on site, we developed an RT-RAA-LFD assay targeting a highly conserved region within the *ORF1b* gene of PDCoV. The diagnostic performance of the RT-RAA-LFD assay in clinical sample detection for PDCoV was evaluated using multiple swine samples via a reference trRT-PCR assay [20].

## Materials and methods

### Viral strains and nucleic acids

PDCoV strain CHN-HN-1601 (GenBank accession No. MG832584.1), porcine epidemic diarrhoea virus (PEDV) strain CHM2013 (GenBank accession No. KM887144.1), porcine enteric alphacoronavirus (PEAV) strain GDZQ-2018 (GenBank accession No. MW727454.1), pseudorabies virus (PRV) strain HB1201 (GenBank accession No. KU057086.1), porcine circovirus type 2 (PCV2) strain BJ-HB (GenBank accession No. AF538325.1), and porcine reproductive and respiratory syndrome virus (PRRSV) strain CHsx1401 (GenBank accession No. KP861625.1) were isolated and preserved in the laboratory. Viral RNAs from transmissible gastroenteritis virus (TGEV) strain Jms and porcine rotavirus (PRoV) strain OSU were generously provided by Prof. Pinghuang Liu of the College of Veterinary Medicine of China Agricultural University, Beijing, China. The cDNA from foot-and-mouth disease virus (FMDV) strain O/BY/CHA/2010 (GenBank accession No. JN998085.1) was kindly provided by Dr. Zixiang Zhu of the Lanzhou Veterinary Research Institute, Lanzhou, China.

### Clinical samples and ethical approval

In 2020–2021, 23 small intestine samples and 40 rectal swabs were obtained from diarrhoeic pigs on commercial pig farms in Guigang City (23°06'N, 109°36” E) in Guangxi Province, Qiannan City (26°27'N, 107°52” E) in Guizhou Province, Yingde City (24°10'N, 113°22” E) in Guangdong Province, and Laizhou City (37°10'N, 119°57” E) in Shandong Province, China. Forty-five serum samples and 41 rectal swabs were collected from piglets on days 1–14 post-inoculation with PDCoV strain CHN-HN-1601 during another experiment conducted in the laboratory. The viral challenge and sample collection procedures were approved by the Laboratory Animal Welfare and Animal Experimental Ethical Committee of China Agricultural University, Beijing, China (No. AW81402202-2-1).

### Nucleic acid extraction

Thirty milligrams of each small intestine sample were thoroughly homogenized in 800 μL phosphate-buffered saline (PBS; pH 7.4) with a PowerLyzer 24 Homogenizer (Qiagen, Hilden, Germany). Each rectal swab sample was thoroughly vortexed with 1 mL PBS. Each sample was centrifuged at 8,000 rpm for 5 min and the RNA was extracted from each 250-μL supernatant with MagZol Reagent (Angen Biotech Co. Ltd., Guangzhou, China) according to the manufacturer’s instructions. Viral RNAs from the PEDV, TGEV, PRoV, PEAV, FMDV, and PRRSV stocks and total RNA from each 250-μL serum sample were extracted with MagZol Reagent. Viral DNAs from PRV and PCV2 were extracted with a TIANamp Virus DNA/RNA Kit (Tiangen Biotech Co. Ltd., Beijing, China) as per the manufacturer’s protocol. Each RNA and DNA extract was eluted in 50 μL nuclease-free water.

### Primer and probe design and synthesis

To determine the optimal PDCoV detection target, 124 currently available genomic PDCoV sequences were downloaded from the GenBank database (https://www.ncbi.nlm.nih.gov/genbank/). The sequences were subjected to multiple sequence alignments with MAFFT v. 7 (https://www.ebi.ac.uk/Tools/msa/mafft/). A   214bp fragment within *ORF1b* was shown to be the most highly conserved region in the PDCoV genome (**Supplementary Figure S1**). Hence, it was selected as the detection target for the RT-RAA-LFD assay. The nfo probe (designated P), seven forward (F1‒F7), and seven reverse (R1‒R7) candidate primers were designed with the aid of Primer Premier v. 5.0 (PREMIER Biosoft, Palo Alto, CA, USA) following the manufacturer’s recommendations. The 5ʹ and 3ʹ ends of the probe were labelled with biotin (antigenic group) and phosphate (polymerase extension blocking group), respectively. The C residue at position 32 of the probe was replaced with a tetrahydrofuran (THF) residue acting as a substrate for exonuclease III cleavage generating a new 3ʹ-hydroxyl group for polymerase extension. Reverse primers were labelled with 6-carboxyfluorescein (FAM; antigenic fluorophore) at their 5ʹ termini. Primer Premier v. 5.0 validated the reliability of the probe/primer sets by detecting the potential formation of cross-dimers between the primers and the probe, self-dimers, and secondary structures (hairpins). An *in silico* specificity check was performed with the NCBI Primer-BLAST web tool (https://www.ncbi.nlm.nih.gov/tools/primer-blast/) to determine whether any primer/probe set could bind a non-target *ORF1b* or a nucleotide paralog in the GenBank database. A primer pair (PD-F/R) and the matching TaqMan probe (PD-Probe) from a previously published trRT-PCR assay for PDCoV [20] were synthesized, and the trRT-PCR assay was conducted as a parallel reference assay. The primers and probes used herein are listed in Table 1 and were commercially synthesized by Sangon Biotech Co. Ltd. (Shanghai, China).

**Table 1.** Primers and probes used in this study.

Primers/Probes	Nucleotide sequences (5′→3′)	Primer/probe position^a^
F1	GTTTAAGCGCTGCGGCTATGAGTATAATGG	16,000‒ 16,029
F2	TGCGGCTATGAGTATAATGGCGTCCATCCA	16,010‒ 16,039
F3	AGTATAATGGCGTCCATCCAGCTCATGCTT	16,020‒ 16,049
F4	CGTCCATCCAGCTCATGCTTTGACCTGGCA	16,030‒ 16,059
F5	GCTCATGCTTTGACCTGGCATGATTGTGGT	16,040‒ 16,069
F6	TGACCTGGCATGATTGTGGTGCAGAGTACC	16,050‒ 16,079
F7	TGATTGTGGTGCAGAGTACCGCTGTGAGGA	16,060‒ 16,089
R1	FAM-GGATACTAGGGTTTTGTATGATATAAGAGT	16,124‒ 16,153
R2	FAM-ACCCAAGTGTGGATACTAGGGTTTTGTATG	16,134‒ 16,163
R3	FAM-GATGGAAGAAACCCAAGTGTGGATACTAGG	16,144‒ 16,173
R4	FAM-AATTTTAAGTGATGGAAGAAACCCAAGTGT	16,154‒ 16,183
R5	FAM-GATATGCATCAATTTTAAGTGATGGAAGAA	16,164‒ 16,193
R6	FAM-AACATATTATGATATGCATCAATTTTAAGT	16,174‒ 16,203
R7	FAM-ACGTGTTAGGAACATATTATGATATGCATC	16,184‒ 16,213
P	Biotin-GGTGCAGAGTACCGCTGTGAGGAGCCACTTG[THF]TAAATTAGTAGGAGT-Phosphorylation	16,067‒ 16,113
mR7	FAM-ACGTGTTAGGAACATATTATGATACGCATC	16,184‒ 16,213
mP	Biotin-GGTCCAGAGTACCGCTGTGAGGAGCCGCTTG[THF]TAAATTAGTAGGAGT-Phosphorylation	16,067‒ 16,113
PD-F	AAAGCTTTCAAGACAATACCT	15,137‒ 15,157
PD-R	TACGACAAACTCCTGAAAGCA	15,203‒ 15,223
PD-Probe	Texas Red-TACGATACGACTGCATTGGCCTAC-BHQ2	15,158‒ 15,181

^a^The location of the primers and probes refers to the whole genome sequence of PDCoV strain CHN-HN-1601 (GenBank accession no. MG832584.1).

Footnote: FAM, 6-carboxyfluorescein; THF, tetrahydrofuran; BHQ2, black hole quencher 2.

### LFD preparation

The lateral flow dipstick consists of a sample pad, a conjugate pad, a nitrocellulose (NC) membrane, and an absorbent pad with adjacent overlapping ends facilitating continuous upward capillary flow of the RAA amplification products. Its working principle and assembly details are shown in Figure 1(b). A sandwich-type detection strategy was employed in the LFD design. The anti-FAM test (T) line captures the PDCoV-specific amplicons conjugated with FAM and biotin. The latter specifically binds to colloidal gold-labelled streptavidin. If the amplification products contain target fragments, the captured complex accumulates on the T line, emerges red, and indicates a positive result. By contrast, the absence of a red signal at the T line suggests a negative result. The conjugate pad was uniformly coated with colloidal gold-labelled streptavidin prepared as previously described [30]. The NC membrane contained the T line sprayed with 4 mg/mL of anti-FAM monoclonal antibody and the control (C) line immobilized with 1.2 mg/mL of bovine serum albumin (BSA)-biotin conjugate. The spraying dose was 1 μL/cm for both lines on the NC membrane. The sprayed NC membrane was dried for 2 h in a dehumidified chamber at 37 °C. It was assembled with the sample and absorbent pads as shown in Figure 1(b). The generated multimembrane composite was cut into 0.5 cm × 5 cm strips with a CM5000 Guillotine Cutter (Biodot Inc., Irvine, CA, USA). The prepared strips were hermetically sealed in plastic bags and stored at room temperature until use.

### Agarose gel electrophoresis-based RT-RAA assay

The RT-RAA assay was conducted in a 25-μL reaction volume using a commercial colloidal gold strip-type RT-RAA kit (Amplification Future, Weifang, China). The reaction mixture consisted of 14.7 μL Buffer A, 1.25 μL Buffer B, 1 μL forward primer (10 μM), 1 μL reverse primer (10 μM), 1 μL RNA template, and 6.05 μL RNase-free ddH_2_O. The reactions were performed in a 40 °C water bath for 10 min. Twenty microlitres of each amplification product were analysed by 2% agarose gel electrophoresis and visualized under ultraviolet light with a Gel Doc™ EZ Imager (Bio-Rad Laboratories, Hercules, CA, USA).

### RT-RAA-LFD assay

The nfo probe-based RT-RAA assay was conducted in a 25-µL reaction volume using the foregoing commercial colloidal gold strip-based RT-RAA kit. The reaction system consisted of 14.7 μL Buffer A, 1.25 μL Buffer B, 1 μL forward primer (10 μM), 1 μL reverse primer (10 μM), 0.3 μL probe (10 μM), 1 μL extracted nucleic acid template, and 5.75 μL RNase-free ddH_2_O. The reaction tubes were briefly centrifuged and placed in a 40 °C water bath for 10 min. After amplification, the RAA products were diluted tenfold with RNase-free water and 50 µL of each diluent was dropped onto the LFD sample pad for visual readout. After 1-min reaction at room temperature, the sample was judged either PDCoV positive when both T and C lines simultaneously appeared or negative when only the C line appeared. The detection result was deemed invalid if any other response was obtained. A positive control and a no-template control (NTC) were included in each reaction set.

### trRT-PCR assay

The *ORF1b*-based trRT-PCR assay for PDCoV detection was performed according to a slightly modified version of a previously described protocol [20]. Briefly, the 25-μL reaction volume comprised 12.5 μL of 2×FastKing one-step probe RT-qPCR master mix (Tiangen Biotech Co. Ltd.), 1 μL of 25×FastKing enzyme mix (Tiangen Biotech Co. Ltd.), 0.625 μL forward primer PD-F (10 μM), 0.625 μL reverse primer PD-R (10 μM), 0.5 μL PD-Probe (10 μM), 2 μL RNA template, and 7.75 μL RNase-free water. The reactions were carried out on a CFX96 real-time PCR thermal cycler (Bio-Rad Laboratories) using the following program: 50 °C for 30 min, 95 °C for 3 min, and 40 cycles of 95 °C for 15 s and 60 °C for 30 s. Samples for which the cycle threshold (Ct) value was <32 were judged positive whereas those with Ct value >35 were judged negative [20].

### Optimization of the RT-RAA-LFD assay reaction conditions

The reaction temperature and reaction time might affect the amplification efficiency of the RT-RAA-LFD assay. Thus, they were optimized using the selected primer-probe combination and the RNA template corresponding to a median tissue culture infectious dose (TCID_50_) of 1.6 × 10^2^ for PDCoV. To optimize the reaction temperature, RT-RAA reactions were conducted at 20 °C, 25 °C, 30 °C, 35 °C, 37 °C, 40 °C and 42 °C for 20 min and each amplification product was tested by LFD. The optical images of the LFD placed on a black plastic sheet in the same position were taken using a Canon EOS 80D digital camera (Tokyo, Japan). Densitometry of the LFD C and T line signal intensities was conducted using ImageJ (National Institutes of Health (NIH), Bethesda, MD, USA). Briefly, the LFD colour images were converted to 8-bit greyscale format using the Image/Type/8-bit command. After eliminating the background signal using the Process/Subtract Background Command with a rolling ball radius value of 50 pixels, the T and C lines on the grey scale image were selected using the ImageJ square selection tool with the same size. The densitometric value of the selected T and C lines was automatically measured, and the output data were subjected to calculate the relative densitometric ratio between the T line and the C line (T/C ratio) for each LFD. The temperature corresponding to the amplification product with the strongest *T*-line signal intensity and the highest T/C ratio was deemed the optimal reaction temperature. At the optimal temperature, each individual RT-RAA reaction was performed for 0, 2, 5, 10, 15, 20, 25 and 30 min. The shortest reaction time required to produce the strongest *T*-line signal intensity and the highest T/C ratio was deemed the optimal reaction time.

### Evaluation of the analytical specificity of the RT-RAA-LFD assay

The nucleic acids extracted from multiple viruses that seriously threaten pig health, namely, PEDV, PRRSV, TGEV, PRoV, PEAV, FMDV, PRV, and PCV2, were detected to evaluate the analytical specificity of the optimized RT-RAA-LFD assay.

### Evaluation of the analytical sensitivity of the RT-RAA-LFD assay

Tenfold serially diluted PDCoV RNA standards equivalent to viral titres in the range of 1.6 × 10^7^–1.6 × 10^˗3^ TCID_50_/mL were used to evaluate the analytical sensitivity of the optimized RT-RAA-LFD assay. To determine the 95% limit of detection (LOD) for the assay, eight independent runs were performed on the RNA standard dilutions. The output data were analysed by Probit regression analysis in SPSS Statistics v. 26.0 (IBM Corp., Armonk, NY, USA). The PDCoV ORF1b-based trRT-PCR assay established by Pan *et al* [20]. was conducted in parallel and its 95% LOD was calculated as described above.

### Repeatability and reproducibility analyses of the RT-RAA-LFD assay

PDCoV RNA (1.6 × 10^7^ TCID_50_/mL, 1.6 × 10^5^ TCID_50_/mL, and 1.6 × 10^3^ TCID_50_/mL) representing strongly, moderately, and weakly positive samples, respectively, were used to assess the repeatability and reproducibility of the RT-RAA-LFD assay. For the inter-assay reproducibility analysis, three independent runs each with three technical replicates were conducted under optimal conditions on the three RNA templates extracted monthly for 3 mo. For the intra-assay repeatability analysis, a single run with nine technical replicates was conducted under optimal conditions on the three RNA templates extracted at a single time point.

### Evaluation of the detection performance of the RT-RAA-LFD assay

Nucleic acids extracted from 23 small intestines, 45 sera, and 81 rectal swabs were detected by the optimized RT-RAA-LFD assay. The detection results were compared against those acquired from the parallel PDCoV *ORF1b*-based trRT-PCR assay [20].

### Statistical analysis

The 95% LOD of the RT-RAA-LFD and trRT-PCR assays were calculated by Probit regression analysis in SPSS Statistics v. 26.0 (IBM Corp.) with the data sets obtained from eight independent runs for each assay. The diagnostic inter-rater agreement between RT-RAA-LFD and trRT-PCR was evaluated by Kappa statistics in SPSS Statistics v. 26.0 with the detection results of 23 small intestine, 45 serum, and 81 rectal swab samples shown in Table 2. Differences were deemed statistically significant when p < 0.05.

**Table 2.** Comparison of the detection performance of the RT-RAA-LFD and trRT-PCR assays for PDCoV detection on 149 clinical swine samples.

Assay name			trRT-PCR			Kappa	P-value
			Positive	Negative	Total		
RT-RAA-LFD	Small intestines	Positive	12	0	12	1.000	<0.001
		Negative	0	11	11		
		Total	12	11	23		
	Rectal swabs	Positive	39	0	39	0.926	<0.001
		Negative	3	39	42		
		Total	42	39	81		
	Sera	Positive	20	0	20	0.955	<0.001
		Negative	1	24	25		
		Total	21	24	45		

## Results

### Design and screening of primer-probe combinations for the RT-RAA-LFD assay

**Figure 2.**
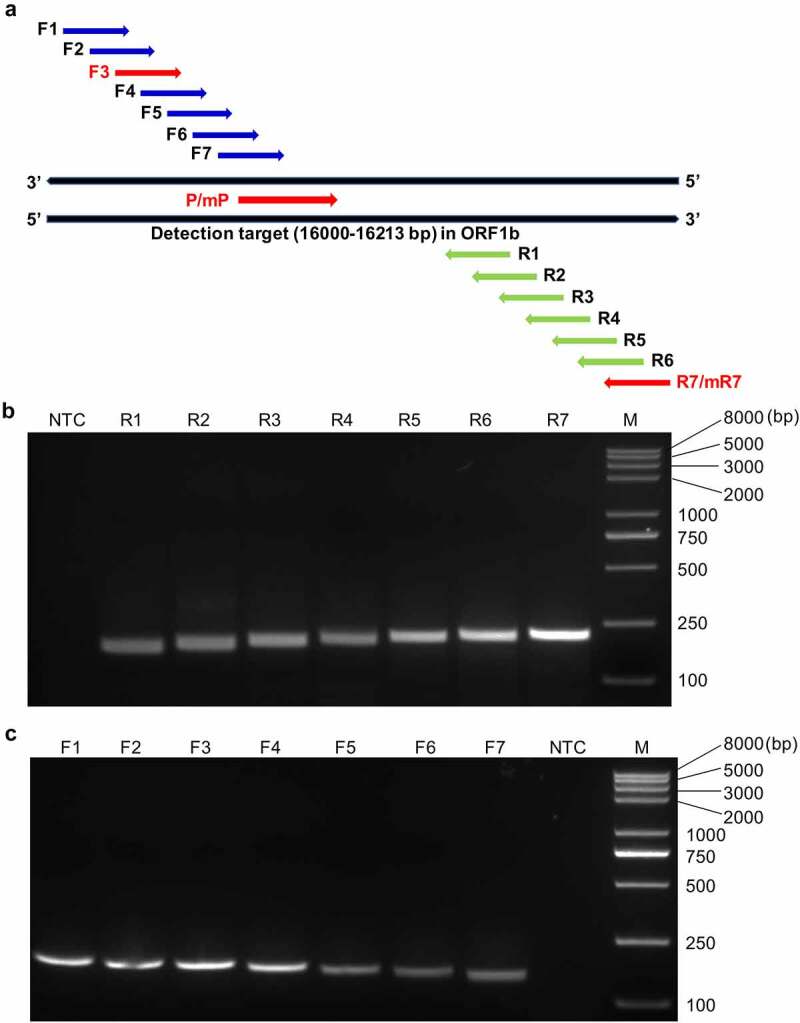
**Design and screening of the optimal primer-probe combinations for the RT-RAA-LFD assay**. (a) Schematic diagram of the relative positions of the candidate primers and nfo probe in the selected detection target (16,000‒16,213 bp) within the *ORF1b* of PDCoV. (b) Representative amplification results of agarose gel electrophoresis-based RT-RAA assays using the randomly selected forward primer F1 to screen all seven reverse candidate primers (R1–R7). (c) Representative amplification results of agarose gel electrophoresis-based RT-RAA assays using R7 to screen all seven forward candidate primers (F1–F7). NTC, no-template control; M, Trans2K Plus II DNA Marker.

We aligned the genomic sequences of the 124 global PDCoV strains available in the GenBank database at the time of study initiation and selected a highly conserved 214-bp fragment within the diagnostic target *ORF1b* (**Supplementary Figure S1**). According to the RAA probe design principles suggested by Amplification Future, and in combination with the Primer Premier v. 5.0 rating, we successfully designed the nfo probe P with the highest score. We designed seven forward (F1‒F7) and seven reverse (R1‒R7) candidate primers surrounding the probe (Figure 2(a) and Table 1). We adopted the screening strategy recommended by the manufacturer to obtain the optimal working primer pair. Briefly, all seven reverse primers were screened with forward primer 1 (F1) and their amplification performance was assessed by the agarose gel electrophoresis-based RT-RAA assay. Figure 2(b) shows that among the seven reverse primers, R7 had the most efficient amplification and the highest product yield followed by R6. Hence, R7 was selected as the optimal reverse primer and used to screen all seven forward primers. It was determined that F3 had the most efficient amplification (Figure 2(c)). Consequently, the combination of F3 and R7 was deemed the optimally performing primer pair for use along with nfo probe P in the subsequent RT-RAA-LFD assays.

### Elimination of false-positive amplification by disrupting primer-probe cross-dimers in the RT-RAA-LFD assay

**Figure 3.**
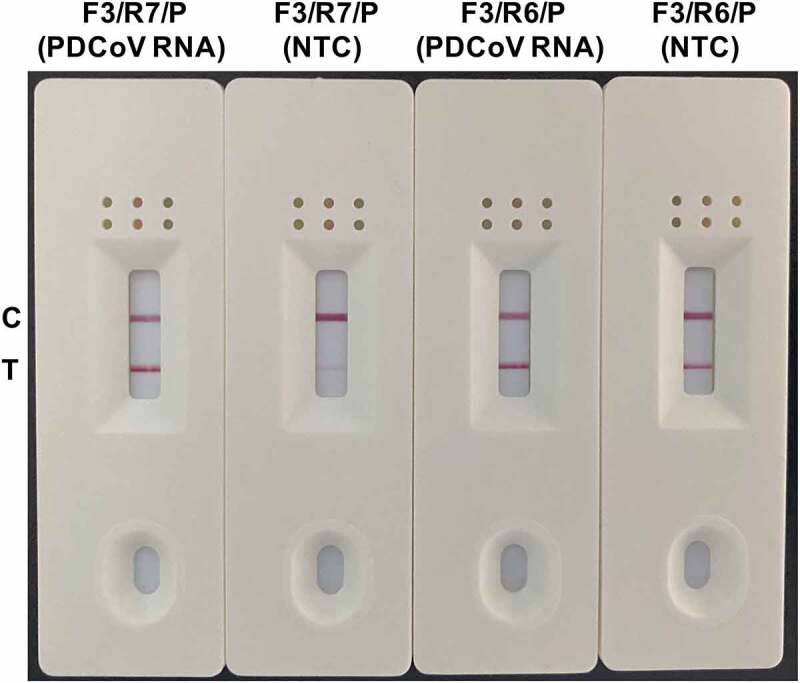
**Performance evaluation of the primer-probe combinations screened for use in the RT-RAA-LFD assay for PDCoV detection**. The amplification performance of the optimized primer-probe combinations F3/R7/P and F3/R6/P was assessed in the RT-RAA-LFD assay using an RNA template corresponding to 1.6 × 10^2^ TCID_50_ PDCoV. A no-template control (NTC) was included in each run. The reactions were performed at 40 °C for 10 min. The amplification products were visualized at room temperature using the prepared LFD. The primer-probe combinations are marked at the top of each strip. C and T represent the control and test lines, respectively.

**Figure 4.**
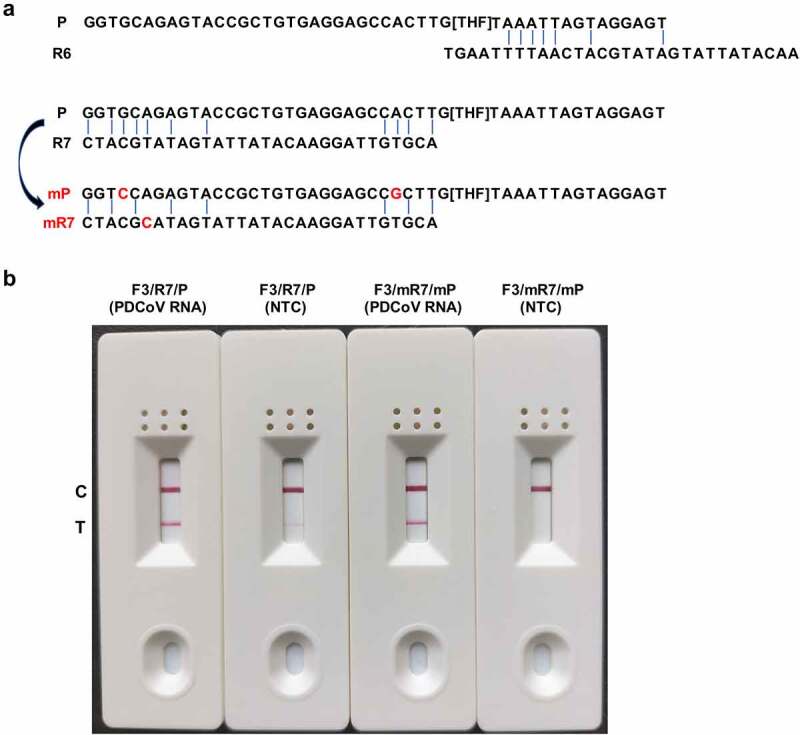
**Elimination of false positives in the RT-RAA-LFD assay by introducing base substitutions into the primer-probe sets**. (a) Matching bases that may be implicated in cross-dimer formation among the nfo probe P, the optimal reverse primer R7, and the suboptimal reverse primer R6 are marked with blue vertical lines. Bases G and A at positions 4 and 27 in the original probe P were mutated to C and G, respectively, and are marked in red in the mutated probe mP. Base T at position 25 in the original reverse primer R7 (corresponding to position 6 [base A] in the reverse complement sequence of R7) was mutated to C and is marked in red in the mutated mR7. (b) the amplification performance of the original primer-probe set F3/R7/P and the optimized primer-probe set F3/mR7/mP was evaluated in the RT-RAA-LFD assay using the RNA template corresponding to 1.6 × 10^2^ TCID_50_ PDCoV. A no-template control (NTC) was included in each run. The amplifications were performed at 40 °C for 10 min. The amplification products were visualized at room temperature using the prepared LFD. The primer-probe sets are indicated at the top of each strip. The control and test lines are denoted on the left side.

To validate the effectiveness of the optimal primer-probe combination (F3, R7, and P) for specific PDCoV detection in the RT-RAA-LFD assay, we conducted an assay on the RNA template corresponding to 1.6 × 10^2^ TCID_50_ PDCoV and included the NTC in all runs. We visualized the amplification products with the prepared LFD. Both PDCoV RNA and the NTC tested positive when F3, R7, and P were used in the RT-RAA-LFD assay (Figure 3). A similar result was obtained using a suboptimal primer-probe combination (F3, R6, and P) (Figure 3). We eliminated the possibility that nucleic acid contamination caused the false positive events. Because the cross-dimer formation between primers and probes has been proven to be an important cause of false positives in nucleic acid isothermal amplification-based lateral flow assays, which might be effectively eliminated by introducing the appropriate base substitutions into the reverse primer and/or probe [31]. Based on this, we chose to use Primer Premier v. 5.0 to analyse matching bases that could form cross-dimers among the nfo probe P, the optimal reverse primer R7, and the suboptimal reverse primer R6. Figure 4(a) shows that the nfo probe P should theoretically generate four consecutive base matches with reverse primer R7 and five consecutive base matches with reverse primer R6. To verify whether these base matches are related to the false positive events in the RT-RAA-LFD assay, we mutated two key bases from G to C and from A to G at positions 4 and 27 of the probe and one key base from T to C at position 25 of the reverse primer R7, respectively. We then re-tested the mutated probe (mP) and reverse primer (mR7) along with the NTC for false positive signals in the RT-RAA-LFD assay. The base mutation strategy was founded on the fact that 5‒9 base mismatches within primer/probe binding regions have no visible effect on the detection performance of RPA/RAA assays [24,32,33]. Figure 4(b) shows that the NTC tested negative in the RT-RAA-LFD assay using the mutated primer-probe combination of F3, mR7, and mP. Hence, the false positive event was successfully eliminated when three base mutations were introduced between the reverse primer R7 and the probe (Figure 4(a)). Based on the T line colour intensity, these base substitutions had no significant effect on the amplification efficiency of RT-RAA-LFD during PDCoV detection (Figure 4(b)). Thus, F3, mR7, and mP comprised the optimal primer-probe combination for the RT-RAA-LFD assay and they were used in the subsequent assays.

### Optimization of the RT-RAA-LFD reaction conditions for PDCoV detection

**Figure 5.**
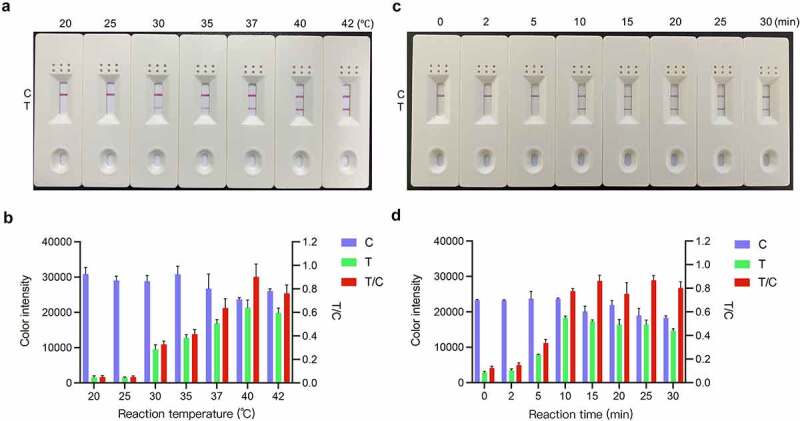
**Optimization of the RT-RAA-LFD assay reaction conditions for PDCoV detection**. (a) Reaction temperature optimization. RT-RAA reactions were run at 20 °C, 25 °C, 30 °C, 35 °C, 37 °C, 40 °C, and 42 °C for 20 min using RNA template corresponding to 1.6 × 10^2^ TCID_50_ PDCoV and the F3/mR7/mP primer-probe combination. The amplification products were visualized at room temperature with the prepared LFD. (b) ImageJ densitometry of the signal intensities at the control (C) and test (T) lines in the LFD and calculation of their relative densitometric ratios. (c) Reaction time optimization. RT-RAA reactions were run at 40 °C for 0 min, 2 min, 5 min, 10 min, 15 min, 20 min, 25 min, and 30 min using RNA template corresponding to 1.6 × 10^2^ TCID_50_ PDCoV and the F3/mR7/mP primer-probe combination. The amplification products were visualized at room temperature with the prepared LFD. (d) ImageJ densitometry of the signal intensities at the C and T lines in the LFD and calculation of their relative densitometric ratios.

The optimal RT-RAA-LFD reaction temperature and time were determined using the RNA template corresponding to 1.6 × 10^2^ TCID_50_ PDCoV. Figure 5(a) shows that although RT-RAA-LFD can operate in the range of 30–42 °C, its amplification efficiency is relatively poor at lower temperatures. At 30 °C and 35 °C, the LFD visual readout was only weakly positive at the T line. At 20 °C and 25 °C, there was no visual readout at the T line. By contrast, RT-RAA-LFD performed well at 37 °C, 40 °C, and 42 °C. Densitometry of the LFD C and T line signal intensities showed that the signal intensity at the T line gradually increased with reaction temperature and reached a maximum at 40 °C (Figure 5(b)). The relative T/C densitometric ratio showed the same trend (Figure 5(b)). Therefore, 40 °C was deemed the optimal RT-RAA-LFD reaction temperature. Under the foregoing optimized conditions, the RT-RAA-LFD assay was conducted for 0 min, 2 min, 5 min, 10 min, 15 min, 20 min, 25 min, and 30 min. Figure 5(c) shows that although a faint T line was visible as early as 5 min after the onset of the RT-RAA-LFD reaction, colour quantification revealed no significant change in the T line colour intensity from 10 min onwards (Figure 5(d)). Based on the detection efficiency, the optimal amplification time was determined to be 10 min. Hence, the optimal reaction conditions for PDCoV detection via our RT-RAA-LFD assay were 10 min at 40 °C for the RT-RAA reaction followed by 1 min at room temperature for the LFD visual readout.

### Evaluation of the specificity of the RT-RAA-LFD assay for PDCoV detection

**Figure 6.**
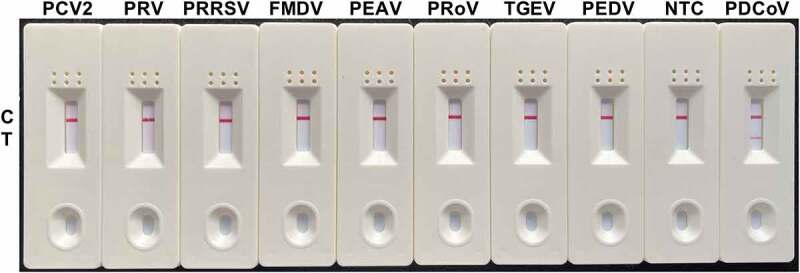
**Specificity analysis of the RT-RAA-LFD assay optimized for PDCoV detection**. Nucleic acids extracted from PEDV, TGEV, PRoV, PEAV, FMDV, PRRSV, PRV, and PCV2 were tested by the optimized RT-RAA-LFD assay at 40 °C for 10 min and using the optimal F3/mR7/mP primer-probe combination. A positive control (1.6 × 10^2^ TCID_50_ PDCoV RNA) and a no-template control (NTC) were included.

Despite a positive reaction to PDCoV, the specificity tests disclosed no positive visual readout for the NTC or any other important swine viruses tested, including PEDV, TGEV, PRoV, PEAV, FMDV, PRRSV, PRV, or PCV2 (Figure 6). The foregoing results suggested that our RT-RAA-LFD assay has good specificity for PDCoV detection.

### Evaluation of the sensitivity of the RT-RAA-LFD assay for PDCoV detection

**Figure 7.**
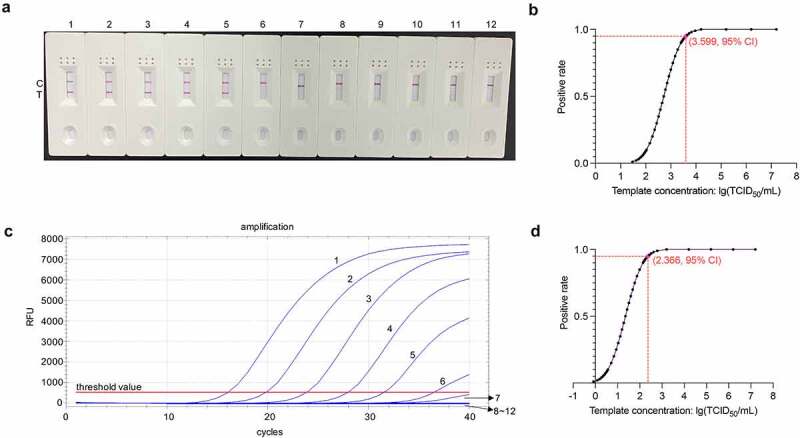
**Sensitivity analysis of the RT-RAA-LFD assay optimized for PDCoV detection**. (a) the optimized RT-RAA-LFD assay was performed to detect tenfold serial PDCoV RNA dilutions. Dipsticks 1 − 12 represent 1.6 × 10^7^–1.6 × 10^˗3^ TCID_50_/mL PDCoV RNA and the no-template control (NTC), respectively. (b) Probit regression analysis in SPSS Statistics v. 26.0 of the RT-RAA-LFD assay of the data for eight independent runs of 1.6 × 10^7^–1.6 × 10 ^−3^ TCID_50_/mL PDCoV RNA dilutions. The 1.6 × 10^7^–1.6 × 10^4^ TCID_50_/mL PDCoV RNA dilutions tested positive in 8/8 runs. The 1.6 × 10^3^ TCID_50_/mL PDCoV RNA dilutions tested positive in 7/8 runs. The 1.6 × 10^2^ TCID_50_/mL PDCoV RNA dilutions tested positive in 1/8 runs. The 1.6 × 10^1^–1.6 × 10^˗3^ TCID_50_/mL PDCoV RNA dilutions tested positive in 0/8 runs. The 95% LOD (10^3.599^ TCID_50_/mL PDCoV RNA/reaction) is depicted by a rhomboid. (c) the *ORF1b*-based trRT-PCR assay was conducted to detect tenfold serial PDCoV RNA dilutions. Curves 1 − 12 represent 1.6 × 10^7^–1.6 × 10^˗3^ TCID_50_/mL PDCoV RNA dilutions and the no-template control (NTC), respectively. (d) Probit regression analysis in SPSS Statistics v. 26.0 of the *ORF1b*-based trRT-PCR assay of the data for eight independent runs of 1.6 × 10^7^–1.6 × 10^˗3^ TCID_50_/mL PDCoV RNA dilutions. The 1.6 × 10^7^–1.6 × 10^3^ TCID_50_/mL PDCoV RNA dilutions tested positive in 8/8 runs. The 1.6 × 10^2^ TCID_50_/mL PDCoV RNA dilutions tested positive in 7/8 runs. The 1.6 × 10^1^ TCID_50_/mL PDCoV RNA dilutions tested positive in 4/8 runs. The 1.6 × 10°–1.6 × 10^˗3^ TCID_50_/mL PDCoV RNA dilutions tested positive in 0/8 runs. The 95% LOD (10^2.366^ TCID_50_/mL PDCoV RNA/reaction) is indicated by a rhomboid.

We determined the LOD for the RT-RAA-LFD assay by using it to assess eleven tenfold serial dilutions of quantified RNA extracted from PDCoV CHN-HN-1601-infected cell culture supernatants. There were eight replicates per RNA dilution. Figure 7(a) shows that the approximate LOD for PDCoV detection by RT-RAA-LFD assay was 1.6 × 10^3^ TCID_50_/mL/reaction. A Probit regression analysis of the datasets obtained from the eight independent runs performed per RNA dilution showed that the 95% LOD for the RT-RAA-LFD assay was 10^3.599^ TCID_50_/mL/reaction (Figure 7(b)). This value is equivalent to 3.97 TCID_50_ PDCoV RNA/reaction. By contrast, the approximate LOD and 95% LOD of the *ORF1b*-based trRT-PCR assay for PDCoV detection were 1.6 × 10^2^ TCID_50_/mL (Figure 7(c)) and 10^2.366^ TCID_50_/mL/reaction (Figure 7(d)), respectively. The latter is equivalent to 0.232 TCID_50_ PDCoV RNA/reaction. The sensitivity of the RT-RAA-LFD assay is slightly lower than that of the trRT-PCR assay. Nevertheless, the RT-RAA-LFD assay has a faster detection speed than the trRT-PCR assay.

### Inter-Assay reproducibility and intra-assay repeatability analyses of RT-RAA-LFD

**Figure 8.**
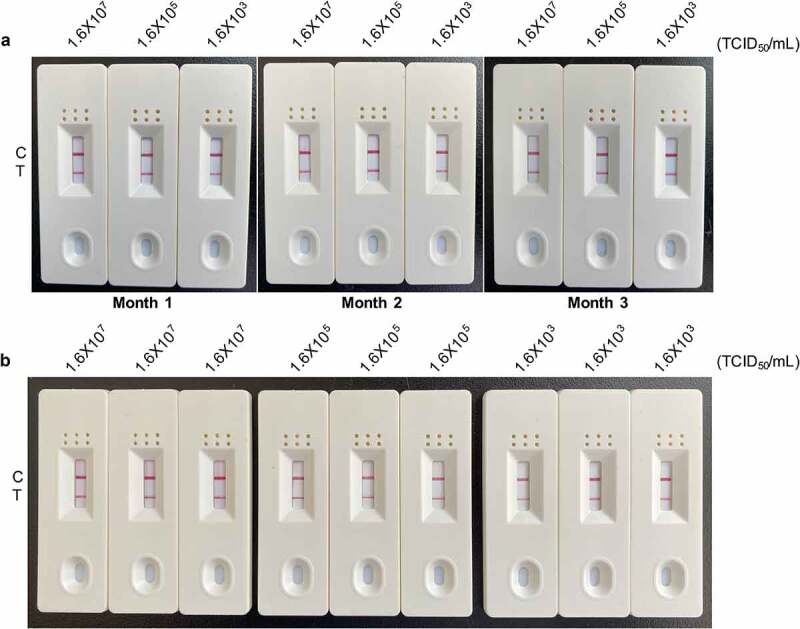
**Reproducibility and repeatability analyses of the RT-RAA-LFD assay optimized for PDCoV detection**. (a) Inter-assay reproducibility test. High, medium, and low PDCoV RNA concentrations (1.6 × 10^7^ TCID_50_/mL, 1.6 × 10^5^ TCID_50_/mL, and 1.6 × 10^3^ TCID_50_/mL) were tested by the optimized RT-RAA-LFD assay in triplicate in three independent runs on RNA templates extracted each month for 3 mo. (b) Intra-assay repeatability test. High, medium, and low PDCoV RNA concentrations (1.6 × 10^7^ TCID_50_/mL, 1.6 × 10^5^ TCID_50_/mL, and 1.6 × 10^3^ TCID_50_/mL) were tested by the optimized RT-RAA-LFD assay as nonuplicate reactions in a single run conducted at a single time point. Control (C) and test (T) lines are labelled on the left side of the dipsticks.

The reproducibility and repeatability of the RT-RAA-LFD assay were evaluated using 1.6 × 10^7^ TCID_50_/mL, 1.6 × 10^5^ TCID_50_/mL, and 1.6 × 10^3^ TCID_50_/mL PDCoV RNA. For the reproducibility assay, the signal intensity at the T line generated by the same PDCoV RNA concentration did not change over 3 mo sampling (Figure 8(a)). Similar results were obtained for the repeatability assay (Figure 8(b)). The latter was conducted using the foregoing RNA concentrations acquired from isolates sampled at a single time point. The preceding data suggested that our RT-RAA-LFD assay has good reproducibility and repeatability in PDCoV detection. Moreover, 3 mo storage had no adverse effect on its amplification performance.

### Validation of the performance of the RT-RAA-LFD assay for PDCoV detection using clinical samples

Twenty-three small intestines, 81 rectal swabs, and 45 sera from swine were simultaneously detected by the RT-RAA-LFD and the *ORF1b*-based trRT-PCR assays [20]. Table 2 shows that both assays returned consistent and comparable detection results for the 23 small intestine samples. However, the assays differed in terms of their detection results for one serum (S16) and three rectal swab samples (RS8, RS27 and RS41). These four samples tested positive for PDCoV by the trRT-PCR assay but negative for PDCoV by our RT-RAA-LFD assay (**Supplementary Figure S2A, B**). The Ct values of the four samples were 34.83, 34.79, 34.84, and 34.97, respectively, and all of these approached the trRT-PCR assay Ct cut-off of <35. To eliminate the possibility of false positive amplification results for the four samples in the trRT-PCR assay, total RNAs were re-extracted from each of them using our previously described nucleic acid enrichment strategy [24]. The enriched RNA was then re-tested by the RT-RAA-LFD and trRT-PCR assays. All four samples were true positives for PDCoV (**Supplementary Figure S2C, D**). Thus, the overall agreement between the RT-RAA-LFD and trRT-PCR assays in terms of clinical detection was 97.32% (145/149). Though both assays were similar and comparable in terms of clinical detection performance, our novel RT-RAA-LFD assay was faster than the traditional trRT-PCR assay.

## Discussion

The newly emerged coronavirus PDCoV has seriously threatened the global pig industry [34] and can infect various avian and mammalian species [10,11,35]. It was recently discovered that PDCoV can also infect humans and has caused an acute undifferentiated febrile illness in children in Haiti [9]. Empirical evidence shows that PDCoV has cross-species transmission ability, zoonotic potential, and a wide host range. It is uncertain whether there exist other unidentified PDCoV hosts or whether the virus will infect new hosts in the future [8,13]. To date, no effective therapeutic agent or commercial vaccine is available for the treatment or prevention of PDCoV infection [34]. Consequently, the development of a simple, rapid, visual diagnostic tool will facilitate timely PDCoV diagnosis, disease management, and subsequent molecular epidemiological investigations. A previous study established that the RPA-LFD assay can rapidly and visually detect PDCoV by targeting the N gene [36]. Nevertheless, the primers and the matching probe were designed based on the limited number of PDCoV genomic sequences then available in the GenBank database. Since that time, however, the number of PDCoV genomic sequences in GenBank has substantially increased. Hence, highly conserved primers and probes can now be designed to develop diagnostic methods that can more comprehensively detect globally distributed epidemic PDCoV strains.

Here, we downloaded 124 whole-genome sequences of representative PDCoV strains derived from China, Korea, Japan, Vietnam, Thailand, Laos, Peru, Mexico, and Haiti. All of these had already been deposited in the GenBank database at the time our study was initiated. Based on multiple sequence alignments, a   214-bp fragment within *ORF1b* was identified as the most conserved region in the entire PDCoV genome (**Supplementary Figure S1**). Therefore, we selected it as the potential diagnostic target to develop our RT-RAA-LFD assay. An *in silico* analysis showed that the *ORF1b*-based primers (F3/R7/mR7) and probes (P/mP) designed for the RT-RAA-LFD assay were considerably more conservative than those designed for the earlier *N*-based PDCoV RPA-LFD assay (data not shown) [36]. We predicted that the performance of our RT-RAA-LFD assay should be superior to that of the RPA-LFD assay in terms of PDCoV detection. As the RPA-LFD assay has not been commercialized, however, we could not compare its detection performance against that of our RT-RAA-LFD assay. We optimized the major reaction conditions of our RT-RAA-LFD assay to improve its detection efficiency. We found that RT-RAA reaction at 40 °C for 10 min and LFD detection at room temperature for 1 min were optimal for PDCoV detection by RT-RAA-LFD. We then used tenfold serially-diluted quantified PDCoV RNA standards to test the analytical sensitivity of our RT-RAA-LFD assay, and compared it against that of the reference trRT-PCR assay for PDCoV [20]. The 95% LOD of RT-RAA-LFD and trRT-PCR were 3.97 TCID_50_ PDCoV RNA/reaction and 0.232 TCID_50_ PDCoV RNA/reaction, respectively. Our RT-RAA-LFD assay was slightly less sensitive than the trRT-PCR assay. Nevertheless, the former had a faster detection speed than the latter.

Another isothermal amplification technique for nucleic acids known as RT-LAMP was also implemented to detect PDCoV [37]. However, our RT-RAA-LFD assay had three major advantages over it. (i) RT-RAA-LFD had a faster detection speed than RT-LAMP. The entire RT-RAA-LFD detection process could be completed within 11 min (10 min at 40 °C for RT-RAA reaction and 1 min at room temperature for visual LFD readout). By contrast, detection by the RT-LAMP assay developed by Zhang et al. required ≥80 min including 70 min at 63 °C for nucleic acid amplification and 10 min at 80 °C for reaction termination [37]. The energy consumption by the assay increased with reaction temperature. (ii) The primer and probe design methods were far simpler for RT-RAA-LFD than RT-LAMP. The former required only one primer pair and one matching probe whereas the latter used four primers to recognize six different regions within the target gene [37]. Primer design complexity is a serious shortcoming of the RT-LAMP assay, which could limit the selection of ideal diagnostic targets for it, and may cause false positive in response to dimer formation among primer sets. (iii) It is more convenient to interpret the detection results of RT-RAA-LFD than those of RT-LAMP. The RT-RAA-LFD amplification products may be conveniently detected by LFD and directly inspected with the unaided eye. By contrast, the detection results of RT-LAMP must be subjected to agarose gel electrophoresis, colorimetry with fluorescent dyes, or restriction enzyme digestion [38,39].

Earlier studies demonstrated that introducing a probe into the RPA/RAA reaction system may augment amplification specificity and diminish primer dimer formation [31,40]. Hence, we designed the nfo probe matching the candidate primer pairs in our RT-RAA-LFD assay. Nevertheless, our trial results showed that the screened primer pair (F3/R7) and the probe (P) produced false positive results in preliminary RT-RAA-LFD detection (Figure 3). It was demonstrated that the false positives produced in nucleic acid isothermal amplification-based lateral flow assays are usually caused by the formation of cross-dimers between primers and probes [31]. For this reason, we used Primer Premier v. 5.0 to identify the bases in the reverse primer R7 and the probe P that could form cross-dimers. It was shown that 5‒9 base mismatches within the primer and/or probe binding regions have no visible effect on the detection performance of RPA/RAA assays [24,32,33]. Primer Premier v. 5.0 predicted that the introduction of single-base substitution into positions 4 (G→C) and 27 (A→G) of probe P and position 25 (T→C) of R7, respectively, could break cross-dimers. A previous study demonstrated that this strategy effectively eliminates cross-dimers formed between the reverse primer and the matching probe [31]. Our results demonstrated that our base substitution strategy was effective and feasible and the mutated reverse primer (mR7) and probe (mP) worked correctly and efficiently in the RT-RAA-LFD assay.

Taken together, the main advantages of our developed RT-RAA-LFD assay include fast detection speed, easy interpretation of detection results, suitability for on-site detection, and low demand for personnel and equipment. However, this assay also has a disadvantage that needs attention during the detection process. After the RT-RAA reaction was completed, it is necessary to open the cap of the reaction tube for LFD detection of the amplification products, which is very easy to cause aerosol pollution in a closed detection environment, thus resulting in false positives.

## Conclusion

The present study established an *ORF1b*-based RT-RAA-LFD assay targeting the most conservative region of the PDCoV genome and validated the efficacy of this assay at rapidly and visually detecting PDCoV-specific nucleic acids. The products amplified by RT-RAA were detected via LFD visualization. Our assay had good analytical sensitivity and specificity and excellent repeatability and reproducibility in PDCoV detection. This assay is rapid and requires neither specialized equipment nor highly qualified personnel. Therefore, it is well suited for on-site PDCoV diagnoses and molecular epidemiological surveys especially in resource-limited or field settings.

## Supplementary Material

Supplemental Material

## Data Availability

All relevant experimental data are available either in the text or in the supplementary materials. Detailed information for PDCoV, PEDV, PDCoV, TGEV, PRoV, PEAV, FMDV, PRRSV, PRV, and PCV2 are available to the public under GenBank accession numbers cited in Materials and Methods section.
